# Treatment for Infertility in Laron Syndrome: A Case Report

**DOI:** 10.7759/cureus.33090

**Published:** 2022-12-29

**Authors:** Elena Alhazidou, Nikolaos Vlachadis, Maria Androulaki, Dimitrios Boudouvas, Georgios Petrakos

**Affiliations:** 1 Department of Obstetrics and Gynecology, General Hospital of Messinia, Kalamata, GRC

**Keywords:** secondary infertility, anovulation, hyperprolactinemia, female infertility, laron syndrome

## Abstract

Laron syndrome is a rare, genetic, growth hormone insensitivity disorder caused by mutations in the growth hormone receptor gene. Affected patients have severe postnatal growth failure, characteristic facial features, and metabolic abnormalities, including severe obesity and metabolic syndrome. Women with Laron syndrome are usually subfertile, mainly due to obesity and metabolic dysregulation, and require treatment for their chronic reproductive dysfunction. To date, infertility in Laron syndrome patients is a rarely addressed problem and, as a result, adequate data regarding its treatment are lacking. Here we present, for the first time in the literature, a rare case of successful treatment of a young woman with Laron syndrome who suffered from infertility due to hyperprolactinemia.

## Introduction

Primary growth hormone insensitivity (GHI), also known as Laron syndrome, is characterized by severe postnatal growth failure. The main characteristic of patients suffering from Laron syndrome is short stature. In addition, a distinctive facial phenotype, obesity, and metabolic syndrome are also observed in these patients. However, it has been shown that affected individuals are at a lower risk of developing cancer and type 2 diabetes [1-5].

The syndrome was first described by Laron in 1966 and is inherited in an autosomal recessive pattern while much less commonly, the condition has an autosomal dominant pattern. The disorder has been reported in only 350 cases worldwide, specifically in the Mediterranean, Middle East, and South Asian regions, including a group living in Ecuador [1,6-7].

A proportion of patients with the syndrome present prolactin hypersecretion, which can lead to anovulation and eventually infertility [1]. In this report, we present the evaluation and successful therapeutic management of an infertile patient with Laron syndrome and hyperprolactinemia.

## Case presentation

The case of a 24-year-old patient of Greek origin, who was diagnosed with Laron syndrome and came to the outpatient clinic of the Obstetrics and Gynecology Clinic of the General Hospital of Messinia due to infertility lasting more than a year, is presented. 

From her family history, both her parents suffered from idiopathic short stature although they had never been tested for chromosomal abnormalities. The patient's medical history included a medical examination at the age of four years due to delayed somatic growth. Laboratory testing was performed with an oral growth hormone (GH) stimulation test with clonidine (determination of serum GH in the morning, in a fasting state, two hours after administration of clonidine at a dose of 0.15 mg/m^2^). The result was marginal (3 ng/ml). Furthermore, the protocol for the insulin-like growth factor-1 (IGF-1) generation test was followed (administration of recombinant human growth hormone at a dose of 33 mcg/kg subcutaneously before bedtime, for four days, and measurement of IGF-1 on day 5). The test result was also non-diagnostic for Laron syndrome (IGF-1 > 15 ng/ml). The results of the other hormonal parameters (prolactin, follicle-stimulating hormone (FSH), luteinizing hormone (LH), and thyroid stimulating hormone (TSH)) were within normal limits. Similarly, the ultrasound thyroid scan and pituitary magnetic resonance imaging (MRI) were normal. Therefore, the delay in the girl's physical growth was classified as idiopathic short stature.

Due to insufficient height gain, the patient was reassessed at seven years of age, but the GH stimulation test with clonidine gave the same marginal result (3 ng/ml), and complete hormonal and imaging tests were again within normal limits.

Menarche occurred at the age of 11 years. At the age of 14 years, the definitive diagnosis of Laron syndrome was made. The patient had extremely low stature (< 5 SD) and positive GH stimulation tests with clonidine and IGF-1. However, the patient was not treated with recombinant IGF-1, because of her bone age, as she had closed epiphyses.

At the age of 24 years, the patient came to our department due to infertility since she had not been able to get pregnant after more than one year of unprotected intercourse with her male partner. The patient's husband's semen parameters were in the normal range. She reported that this was her first partner and that she had never been pregnant before. The patient reported spontaneous menarche at the age of 13 years, and afterward, she had regular menses for about seven years. However, she reported some months of oligomenorrhea during the last four years. Transvaginal ultrasound and MRI were performed, with normal imaging of the internal genitalia and the hypothalamic-pituitary tract. Hormonal test results revealed low levels of FSH, LH, and estradiol, and elevated prolactin (Table 1).

**Table 1:** Hormonal test results at the time of diagnosis FSH: follicle-stimulating hormone; LH: luteinizing hormone

Test	Result	Normal range
FSH (U/l)	2.4	3.5-12.5
LH (U/l)	1.3	2.4–12.6
Estradiol (pg/ml)	21	25-156
Prolactin (ng/ml)	92	5-25

The patient was started on cabergoline (a dopamine D2 receptors agonist), at a dose of 0.25 mg, twice a week. Prolactin levels returned to normal (<25 ng/ml) after three months, and treatment was continued for a further six months. The levels of gonadotropins and estradiol returned to normal and ovulation was repeatedly confirmed hormonally (mid-luteal phase serum progesterone levels >10 ng/ml) and ultrasonographically (by the appearance and disappearance of a dominant follicle > 14 mm on transvaginal ultrasound).

The patient conceived spontaneously three months after discontinuation of cabergoline treatment. It was an uncomplicated, full-term pregnancy and a healthy male neonate was born vaginally with a birth weight of 2,300 g, length of 47 cm, head circumference of 31 cm, and Apgar score of 9/10.

## Discussion

This article presents the case of a young woman with Laron syndrome and infertility, which was successfully treated. 

Laron syndrome is a very rare genetic disease, with an estimated prevalence of 1-9/1,000,000. The disorder is a result of mutations affecting the GH receptor gene. The most common mutations affect the extracellular binding region of the receptor. However, mutations can also occur in its downstream mediators, resulting in GH resistance and the loss of IGF-1 synthesis [2]. Clinically, patients might be indistinguishable from children suffering from isolated GH deficiency; however, Laron syndrome patients are usually identified by normal or high levels of GH and low levels of IGF-1 [7].

Studies have shown hyperprolactinemia may be present in patients with Laron syndrome. The etiology of this phenomenon can be found in the positive feedback to the IGF-1 deficiency, resulting in the augmentation of GH secretion. When GH is oversecreted, prolactin is usually also found in high levels, possibly due to their common origin, as a result of a drift phenomenon of the mammosomatotrophs [8]. Hyperprolactinemia leads to amenorrhea, as the elevated prolactin levels suppress gonadotrophin-releasing hormone (GnRH) secretion and therefore inhibit LH and FSH secretion [9].

Evaluation of Laron syndrome patients can be complicated. Children presenting the typical phenotype, especially growth retardation, defined as height < -3 standard deviation (SD), together with a high concentration of GH (basal GH > 2.5 ng/ml) and low level of IGF-1 in serum (basal IGF-1 < 50 mcg/l) should be evaluated. After excluding various conditions that can possibly influence both GH and IGF-1 secretion, such as malnutrition, hepatic disease, or hypothalamic-pituitary insufficiency, the IGF-1 generation test should be performed. Other suggestive findings include low levels of insulin-like growth factor binding protein-3 (IGFBP-3) (< 2 SD) and GH binding (< 10%). Results of the IGF generation test are positive if IGF-1 < 15 mcg/L and/or IGFBP-3 < 0.4 mg/L. Diagnosis of Laron syndrome requires five of the seven abovementioned parameters to be positive [10]. Finally, the diagnosis should be confirmed with molecular genetic testing, by sequencing the GH receptor gene [11].

The IGF-1 generation test has been the basic tool for establishing the diagnosis of Laron syndrome for children presenting growth failure. However, this test is often inadequate, as happened in the presented case. Our patient had to undergo an IGF-1 generation test three times, at three different ages (4, 7, and 14 years old) until diagnosis was made. It is known that there are certain limitations of the IGF-1 generation test in children with short stature [12]. Specifically, it is suggested that the specificity and sensitivity of the IGF-1 generation test are not high enough to constitute a reliable GHI syndrome diagnosis, especially in children suffering from the severe form. Another tool usually used in the investigation of children with short stature, the clonidine stimulation test, also used in our patient’s case, also presents low diagnostic value, especially in children with elevated body mass index. Taking into account that obesity is a common clinical feature of Laron syndrome patients, it becomes apparent that the clonidine stimulation test presents poor reliability in these patients [13]. Molecular sequencing of the GH receptor gene presents the best diagnostic value and should be performed at a young age in order so that the treatment with recombinant IGF-1 can be administered before epiphyses closure.

Several women with Laron syndrome may experience infertility, as in the case presented here, where infertility was attributed to hyperprolactinemia. Hyperprolactinemia alters the pulsatile secretion of GnRH, affecting the release of LH and FSH, and therefore leads to anovulation and/or amenorrhea. The therapeutic goal was the treatment of hyperprolactinemia, however, there is insufficient relevant clinical data. Dopamine agonists are the cornerstone for the treatment of hyperprolactinemia, as they stabilize prolactin levels to normal and restore ovulation [14]. Cabergoline is largely used in the treatment of hyperprolactinemia and was selected at a dose of 0.5 mg/week (specifically 0.25 mg twice a week) [15-16]. This therapeutic option proved to be effective, as it led to the normalization of prolactin levels and, eventually, to spontaneous pregnancy. To our knowledge, this is the first published report of hyperprolactinemia restoration and spontaneous successful pregnancy in a Laron syndrome patient. It remains to be further studied whether this treatment would be equally adequate for other patients suffering from the same disorder and at what dose, depending on prolactin or GH levels. In addition, it would be interesting to evaluate bromocriptine, another dopamine agonist, as a treatment for hyperprolactinemia in patients with Laron syndrome in order to achieve fertility. To date, treatment of Laron syndrome has focused on achieving optimal growth potential with the administration of recombinant IGF-1 and data are lacking regarding the correction of hyperprolactinemia and restoration of ovulation and fertility in these patients.

## Conclusions

Laron syndrome is a rare condition with numerous clinical and endocrinological characteristics. The presented case reveals the inadequacy of clinical data available at present for the diagnosis of GHI syndromes and illustrates the necessity of adopting more reliable strategies. Furthermore, one of the common clinical manifestations of the syndrome is infertility, which is usually associated with the metabolic effects of obesity. In the case presented, a young patient with Laron syndrome achieved a full-term pregnancy after medical correction of hyperprolactinemia. This case contributes to the literature by demonstrating that treatment with cabergoline was efficient to normalize prolactin levels and attain spontaneous conception. Nevertheless, the therapy of choice for Laron syndrome patients in order to achieve fertilization constitutes a domain needing further investigation.
